# Biofilm destruction activity of α-tocopherol against *Staphylococcus aureus, Proteus mirabilis*, and *Pseudomonas aeruginosa*

**DOI:** 10.1093/femsle/fnaf020

**Published:** 2025-02-04

**Authors:** Pui Yee Leong, Wei Qi Tan, Wee Sim Choo

**Affiliations:** School of Science, Monash University Malaysia, Jalan Lagoon Selatan, 47500 Bandar Sunway, Selangor, Malaysia; School of Science, Monash University Malaysia, Jalan Lagoon Selatan, 47500 Bandar Sunway, Selangor, Malaysia; School of Science, Monash University Malaysia, Jalan Lagoon Selatan, 47500 Bandar Sunway, Selangor, Malaysia

**Keywords:** anti-biofilm, antimicrobial, biofilm inhibition, Gram-negative bacteria, Gram-positive bacteria, vitamin E

## Abstract

Antibiotic resistance and the persistence of sessile cells within biofilms complicate the eradication of biofilm-related infections using conventional antibiotics. This highlights the necessity for alternate therapy methods. The objective of this study was to investigate the biofilm destruction activity of α-tocopherol against *Staphylococcus aureus, Proteus mirabilis*, and *Pseudomonas aeruginosa* on polystyrene. α-Tocopherol showed significant biofilm destruction activity on the pre-formed biofilms of *S. aureus* (45%–46%), *Pr. mirabilis* (42%–54%), and *Ps. aeruginosa* (28%). Resazurin assay showed that α-tocopherol disrupted all bacterial biofilms without interfering with their cell viability. Scanning electron microscope images showed lower bacterial cell count and less compacted cell aggregates on polystyrene surfaces after treatment with α-tocopherol. This study demonstrated the biofilm destruction activity of α-tocopherol against *S. aureus, Pr. mirabilis*, and *Ps. aeruginosa*. α-Tocopherol could potentially be used to decrease biofilm-associated infections of these bacteria.

## Teaser

α-tocopherol, a member of the vitamin E family, exhibits biofilm destruction activity against pathogenic bacteria such as *Staphylococcus aureus, Proteus mirabilis*, and *Pseudomonas aeruginosa*.

## Introduction

There are two major states of bacteria: the planktonic and biofilm states, where bacteria exist predominantly in biofilm (Kang et al. 2012, Seth et al. 2012). The adherent bacteria in biofilms differ profoundly from their planktonic counterparts in their intrinsic defense and survival mechanisms (Cos et al. 2010, Seth et al. 2012). It is estimated that ~68% of human microbial infections are associated with bacteria biofilms, as they can colonize living surfaces such as host tissue or non-living surfaces such as surfaces of medical implants (Crouzet et al. 2014, Hong et al. 2014, Jamal et al. 2018, Ghosh et al. 2020). Biofilm has been implicated as an independent contributor that impedes wound healing in patients with chronic wound infections, where the bacteria can colonize the wound surface through biofilm formation (Seth et al. 2012). The sessile bacteria in biofilm often cause chronic infections as they are tolerant to conventional antimicrobials. Even in healthy individuals, biofilm infections can rarely be resolved until the biofilm is eradicated (Cos et al. 2010). As a result of the chronicity, resistance to antimicrobial therapies, and immunological evasion ability of biofilm, clinical biofilm-related infections in humans often lead to significant morbidity (Hernández-Jiménez et al. 2013, Suresh et al. 2019). This further complicates and limits the therapeutic options in the management of biofilm infections in humans (Seth et al. 2012, Hernández-Jiménez et al. 2013). As several resistance mechanisms of biofilms are different from the resistance mechanisms of planktonic bacteria to antimicrobial agents, an increase in resistance of biofilms can occur if these resistance mechanisms contribute together (Yong et al. 2019a).

*Staphylococcus aureus* is one of the most frequently found opportunistic pathogens in biofilm-associated nosocomial infections, including medical device-related infections (Lister and Horswill 2014, Mawang et al. 2017, Kong et al. 2018, Suresh et al. 2019). The staphylococcal species is accountable for ~70% of implantable device-associated infections, with the majority of these infections being associated with *S. aureus* (Moormeier and Bayles 2017, Khatoon et al. 2018). As *Proteus mirabilis* has the ability to form crystalline biofilms on the surface of the catheter upon chemical reaction with urine, this makes it frequently associated with catheter-associated urinary tract infections in humans. The bacteria embedded in the crystalline biofilms are highly recalcitrant to the human immune system and many conventional antibiotics (Wasfi et al. 2020). *Pseudomonas aeruginosa* usually establishes itself in immunocompromised patients, such as patients with cystic fibrosis or patients in intensive care units in hospitals (de bentzmann and Plésiat 2011). *Pseudomonas aeruginosa* can also cause several chronic wound infections similar to *S. aureus*, including chronic leg ulcers, skin, and diabetic ulcers. About 52% of patients with chronic leg ulcers are colonized by *Ps. aeruginosa*, where the virulence factor expression of the bacteria is increased, leading to severe infections and antibiotic resistance (Crouzet et al. 2014, Serra et al. 2015). These three pathogens share the same characteristic where they are the common opportunistic pathogens for biofilm-related infections in the human body.

The occurrence of antibiotic resistance of bacteria in biofilm and the persistence of sessile cells have necessitated the development of effective alternative treatments for biofilm-associated infections. Antimicrobials derived from plants have more structural and biochemical diversity than synthetic drugs. It would be more difficult for the bacteria to interact with the high complexity of natural compounds, and this could contribute to the development of alternative therapies to treat biofilm-related infections (Roy et al. 2018). Tocopherols are the major vitamin E components in most edible oils, such as sunflower and olive oils (Shahidi and de Camargo 2016). Tocopherol has been shown to reduce bacterial adherence of some *Staphylococcus epidermidis* and *S. aureus* strains on ultra-high molecular weight polyethylene (UHMWPE; Gómez-Barrena et al. 2011), reduce bacterial adhesive ability of *S. aureus* and *Escherichia coli* on various types of UHMWPE (Banche et al. 2014), reduce bacterial adhesion and biofilm accumulation of *S. epidermidis* and *S. aureus* on polylactic acid (Campoccia et al. 2015), and prevent bacterial adherence and biofilm formation of *S. aureus* on a cross-linked polyethylene surface with a poly(2-methacryloyloxyethyl phosphorylcholine) layer (Kyomoto et al. 2015). Biofilm formation of *S. aureus, S. epidermidis, E. coli, Klebsiella pneumoniae, Pr. mirabilis, Acinetobacter baumannii, Ps. aeruginosa*, and *Ps. putida* has been shown to reduce with α-tocopheryl acetate application on polystyrene (Vergalito et al. 2015). Vitamin E reduced 62% and 67% of the biofilm formation of *Ps. aeruginosa* PAO1 in a flat-bottomed 96-well plate (Soltani et al. 2021) and a molecular imprinting polymer (Tajani et al. 2022), respectively. The objective of this study was to investigate the biofilm destruction activity of α-tocopherol against *S. aureus, Pr. mirabilis*, and *Ps. aeruginosa* on polystyrene.

## Materials and methods

### Bacterial strains

Cultures of *S. aureus* ATCC 6538P, *Pr. mirabilis* ATCC 12453, and *Ps. aeruginosa* ATCC 27853 were obtained from American Type Culture Collection (ATCC) Manassas, VA, USA.

### Preparation of α-tocopherol solution

α-Tocopherol was prepared by dissolving 4 mg of α-tocopherol ($\ge $96%, Sigma–Aldrich, Steinheim, Germany) in 10 µl of dimethyl sulfoxide (DMSO; Calbiochem, CA, USA), followed by 990 µl of brain–heart infusion (BHI) broth to achieve a final concentration of 4 mg ml^−1^ and subjected to 45 s of sonication.

### Antimicrobial test

An antimicrobial test using the broth microdilution method was conducted to determine the minimum inhibitory concentration (MIC) of a sample (Clinical and Laboratory Standards Institute 2012). MIC is the lowest concentration of a compound that will cause inhibition of the visible growth of a microorganism. The bacteria were incubated in 10 ml BHI broth (Oxoid, Hampshire, UK) at 37°C for 18 h. Then, the bacteria suspension was adjusted to 0.5 McFarland standard using BHI broth (absorbance of 0.08–0.11 at 625 nm), equivalent to ~1 $\times $ 10^8^ colony-forming unit (CFU) per ml. The adjusted bacterial suspension was diluted 1:1000 in sterile BHI broth (equivalent to ~1 $\times $ 10^5^ CFU ml^−1^). α-Tocopherol solution (100 µl) was serially diluted into 96-well microtiter plates with BHI broth before inoculation of 100 µl of diluted bacterial suspension (the highest concentration of α-tocopherol will be 2 mg ml^−1^ after inoculating with bacteria suspension). The concentration of DMSO in the wells was 0.5% DMSO. After 24 h incubation at 37°C, the absorbance was measured at 625 nm using a microplate reader (Tecan, Männedorf, Switzerland). The negative control (NC) was a bacterial suspension in BHI broth with 0.5% DMSO, and the positive control (PC) was a bacterial suspension in BHI broth with chloramphenicol (Nacalai Tesque, Kyoto, Japan). Chloramphenicol (100 mg ml^−1^) was first prepared by dissolving 20 mg of chloramphenicol in 200 µl of absolute ethanol, followed by taking 10 µl of this solution and adding 990 µl of BHI broth to achieve the starting concentration of 1 mg ml^−1^ to be used for serial dilution.

### Crystal violet assay

Crystal violet assay was carried out to determine the biofilm destruction activity of a sample (Jeyaraj et al. 2022). This assay allowed the determination of the minimum biofilm destruction concentration (MBDC) of a sample. MBDC is the lowest sample concentration that can destroy a pre-formed biofilm. The bacteria were incubated in BHI broth at 37°C for 18 h. Then, the bacteria suspension was adjusted to 0.5 McFarland standard. The bacterial suspension was then diluted 1:100 (equivalent to ~1 × 10^6^ CFU ml^−1^) in BHI broth supplemented in 1% glucose (Oxoid, Hampshire, UK) to stress the biofilm formation by bacteria. To determine biofilm destruction activity, diluted bacterial suspension (200 µl) was inoculated into 96-well microtiter plates. This was followed by incubation at 37°C for 24 h to allow biofilm formation. Then, the broth in the wells was removed, and the plate was rinsed gently with sterile phosphate-buffered saline (PBS; Oxoid, Hampshire, UK) three times. Next, a two-fold serial dilution of the α-tocopherol solution (200 µl) was added to the wells accordingly. The concentration of DMSO in the wells was 0.5% DMSO. The plate was incubated for 24 h at 37°C. Then, the contents of the wells were removed, and the wells were rinsed with water gently three times. The wells were heat-fixed at 60°C for 1 h, followed by staining with 0.2 ml of 0.1% crystal violet for 10 min. The wells were then rinsed with water and air-dried. Lastly, 0.2 ml of 95% ethanol was added, and absorbance was measured at 570 nm. The NC was bacteria suspension in BHI broth with 0.5% DMSO, while the PC was bacteria suspension in BHI broth with 1% sodium hypochlorite. Results were expressed as % destruction = [(absorbance of NC at 570 nm − absorbance of treated sample at 570 nm)/absorbance of NC at 570 nm] $\times $ 100.

### Resazurin assay

This assay allowed the evaluation of the effect of a substrate on the cellular viability of bacterial cells within a biofilm matrix (Toté et al. 2008). The bacteria were incubated in BHI broth at 37°C for 18 h. Next, the bacteria suspension was adjusted to 0.5 McFarland standard. Then, the bacterial suspension was diluted 1:100 in BHI with 1% glucose. The diluted bacterial suspension (200 µl) was inoculated into wells and incubated for 24 h at 37°C to allow biofilm formation. The broth was discarded after 24 h of incubation, and the adherent cells in the wells were washed with sterile PBS two times before adding a two-fold serial dilution of the α-tocopherol solution (200 µl) followed by incubation for 24 h at 37°C. The concentration of DMSO in the wells was 0.5% DMSO. The NC was bacteria suspension in BHI broth with 0.5% DMSO, while the PC was bacteria suspension in BHI broth with 1% sodium hypochlorite. The plates were rinsed with sterile distilled water before adding 5 µg ml^−1^ of resazurin (Sigma–Aldrich, Steinheim, Germany) in sterile distilled water. The plate was incubated for 2 h at 37°C. After the incubation, the fluorescence intensity (*λ*_ex_: 560 nm and *λ*_em_: 590 nm) was measured. Results were expressed as % cell viability = fluorescence units of treated sample/fluorescence units of NC $\times $ 100.

### Scanning electron microscopy

Scanning electron microscopy (SEM) was used to observe bacterial cell density and distribution of biofilm after α-tocopherol treatment at MBDC (Yong et al. 2021). Polystyrene discs with dimensions of 1 cm $\times $ 1 cm were cleaned by soaking them in 70% ethanol for 5 min. This was followed by rinsing them with sterile distilled water and placing them in a clean Petri dish. The bacteria were incubated in BHI broth at 37°C for 18 h. Next, the bacteria suspension was adjusted to 0.5 McFarland standard (absorbance of 0.08–0.11 at 625 nm), equivalent to ~1 × 10^8^ CFU ml^−1^ in BHI broth. Then, the bacterial suspension was diluted 1:100 in BHI with 1% glucose. The adjusted bacterial suspension was added to a 24-well microtiter plate and incubated for 24 h at 37°C. Then, the discs were washed gently with sterile PBS and placed into a new 24-well microtiter plate inoculated with a sample at MBDC. Incubation for 24 h at 37°C was carried out. Then, the discs were washed gently with sterile PBS. The discs were prepared for SEM according to the following procedures: fixing with 2.5% (v/v) glutaraldehyde in sterile PBS for 4 h at room temperature and soaking in sterile PBS for 10 min afterward. Ethanol dehydration was carried out using ethanol with concentrations of 20, 40, 60, 70, 80, 90, 95, and 100% (v/v) in sterile PBS sequentially with 10 min for each concentration. The discs were kept in a desiccator overnight. The surfaces of the discs were gold-sputtered using a sputter coater (Quorum, Laughton, UK) before examination using a scanning electron microscope (Hitachi S-3400N-II, Chiyoda, Japan). Images were taken under 2500× magnification at 10 kV. Bacterial cell numbers were estimated using Fiji (distribution of the ImageJ software, US National Institutes of Health, Bethesda, USA).

### Statistical analysis

Antimicrobial test, crystal violet assay, and resazurin assay were carried out in independent triplicates, whereas SEM was carried out in duplicates. Then, all data were analysed using one-way ANOVA. Post hoc Tukey’s test was carried out at *P* < .05.

## Results

**Figure 1.**
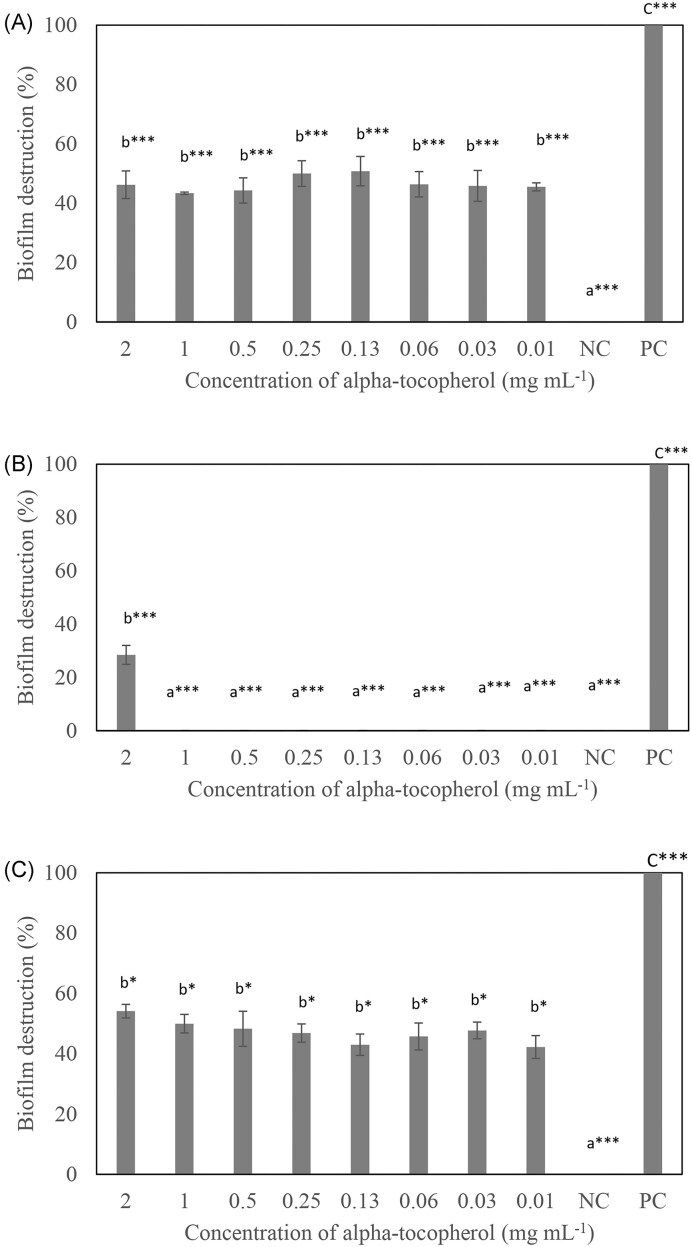
Biofilm destruction after treatment with α-tocopherol at various concentrations against (A) *S. aureus* ATCC 6538P, (B) *Ps. aeruginosa* ATCC 27853, and (C) *Pr. mirabilis* ATCC 12453. The NC was bacteria suspension in BHI broth with 0.5% DMSO water, while the PC was bacteria suspension in BHI broth with 1% sodium hypochlorite. All experiments were carried out in triplicates, and results were expressed as mean percentage biofilm destruction ± standard deviation. ^abc^Different letters indicate significant differences in biofilm destruction at ***P < .001 or *P < .05.

**Figure 2.**
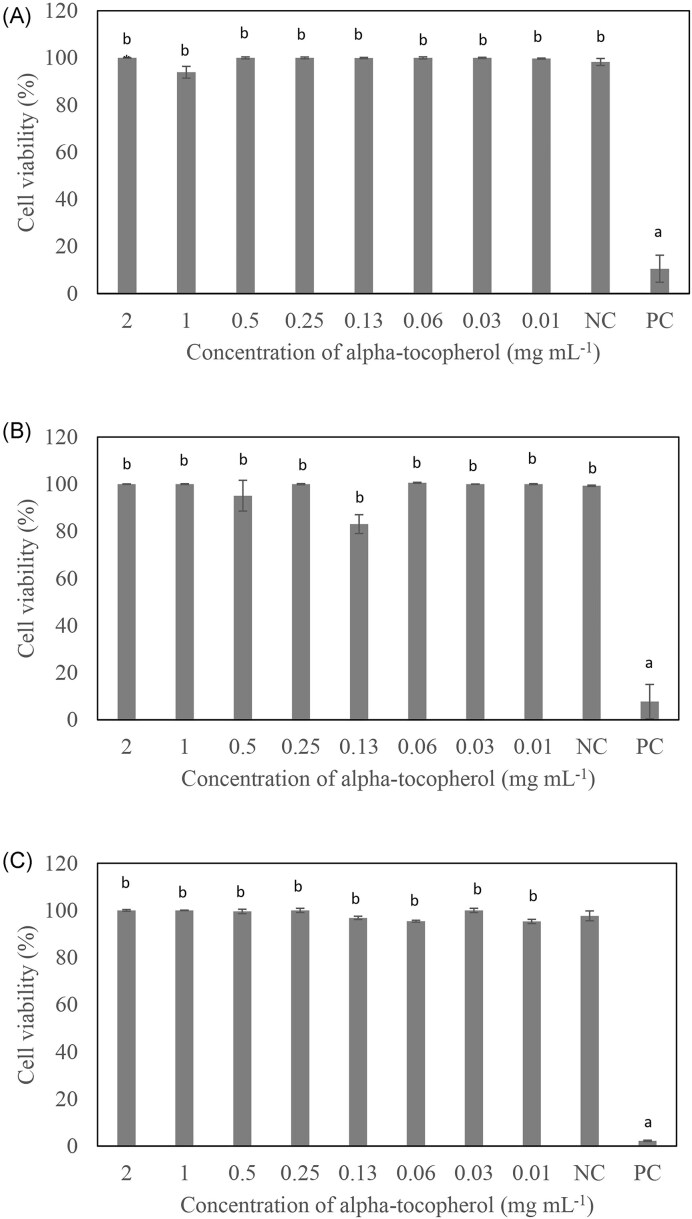
Cell viability within bacterial biofilms after treatment with α-tocopherol at various concentrations against (A) *S. aureus* ATCC 6538P, (B) *Ps. aeruginosa* ATCC 27853, and (C) *Pr. mirabilis* ATCC 12453. The NC was bacteria suspension in BHI broth with 0.5% DMSO, while the PC was bacteria suspension in BHI broth with 1% sodium hypochlorite. All experiments were carried out in triplicates, and results were expressed as mean percentage cell viability ± standard deviation. ^ab^Different letters indicate significant differences in cell viability at *P* < .001.

The MIC values of α-tocopherol were more than 2 mg ml^−1^ for *S. aureus* ATCC 6538P, *Pr. mirabilis* ATCC 12453, and *Ps. aeruginosa* ATCC 27853 (Table 1). α-Tocopherol was able to disrupt the biofilms formed by *S. aureus* ATCC 6538P (45%–46%) and *Pr. mirabilis* ATCC 12453 (42%–54%) at all the concentrations of α-tocopherol tested, whereas it was only effective in disrupting *Ps. aeruginosa* ATCC 27853 biofilm at 2 mg ml^−1^ (28%) to a lower extent compared to the other two bacteria tested (Fig. 1). The MBDC of α-tocopherol was determined to be 0.01 mg ml^−1^ for *S. aureus* ATCC 6538P and *Pr. mirabilis* ATCC 12453, and 2 mg ml^−1^ for *Ps. aeruginosa* ATCC 27853. α-Tocopherol was shown to have no significant effect on the cell viability of *S. aureus* ATCC 6538P, *Pr. mirabilis* ATCC 12453, and *Ps. aeruginosa* ATCC 27853 from 0.01 to 2 mg ml^−1^ (Fig. 2).

**Table 1.** MIC of α-tocopherol against *S. aureus* ATCC 6538, *Pr. mirabilis* ATCC 12453, and *Ps. aeruginosa* ATCC 27853.

Bacteria strains	α-Tocopherol MIC (mg ml^−1^)	Chloramphenicol MIC (mg ml^−1^)
*Staphylococcus aureus* ATCC 6538P (MSSA)	>2	0.008
*Proteus mirabilis* ATCC 12 453	>2	0.004
*Pseudomonas aeruginosa* ATCC 27 853	>2	0.250

All experiments were carried out in independent triplicates.

**Figure 3.**
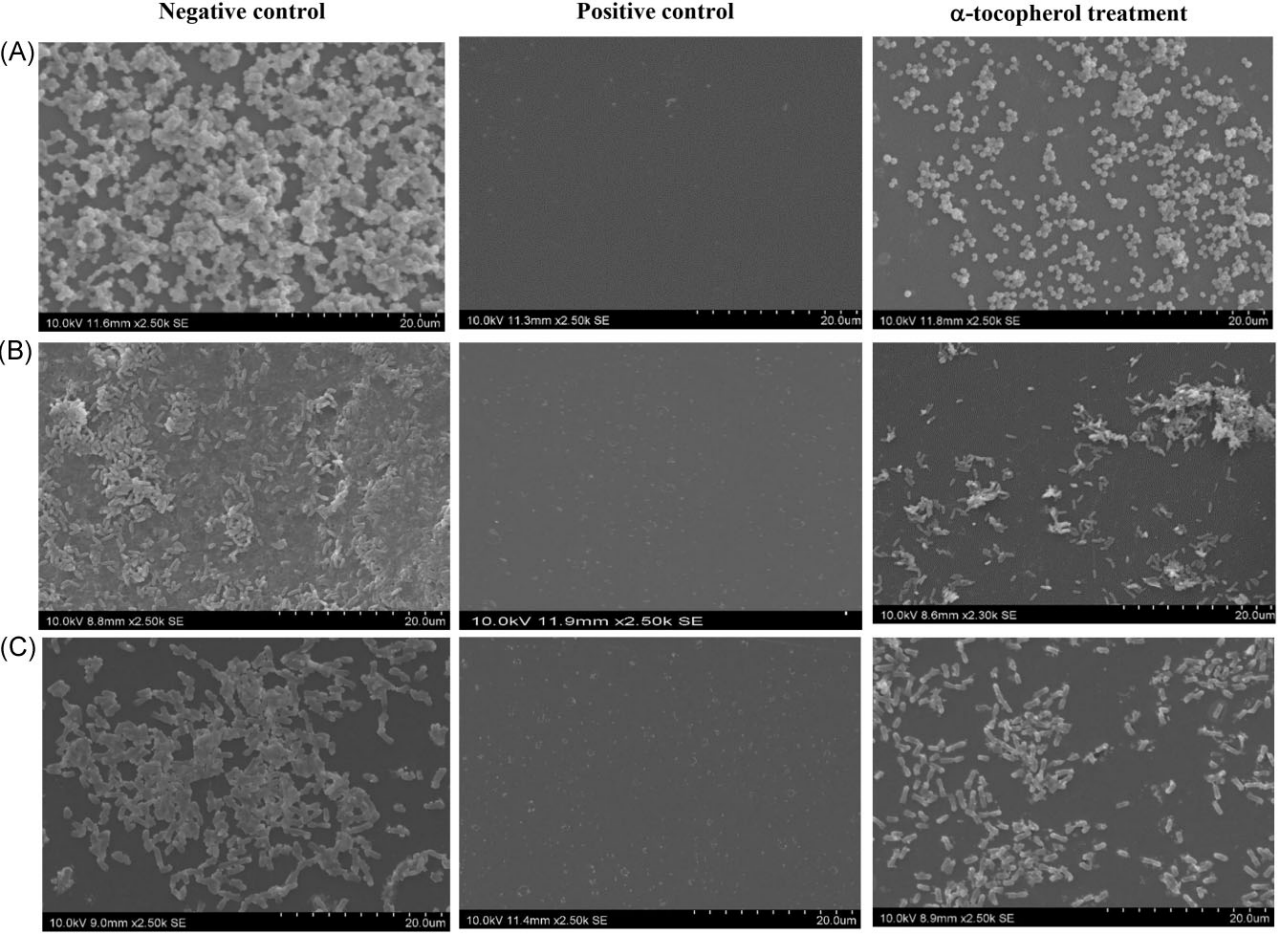
Effect of α-tocopherol on the bacterial cell density and distribution of biofilm after treatment with α-tocopherol at MBDC for (A) *S. aureus* ATCC 6538P, (B) *Ps. aeruginosa* ATCC 27853, and (C) *Pr. mirabilis* ATCC 12453. Images were taken under 2500× magnification at 10 kV. The NC was bacteria suspension in BHI broth with 0.5% DMSO. The PC was bacteria suspension in BHI broth with 1% sodium hypochlorite. The MDBC was 0.01 mg ml^−1^ for *S. aureus* ATCC 6538P and *Pr. mirabilis* ATCC 12453, and 2 mg ml^−1^ for *Ps. aeruginosa* ATCC 27853.

The effect of α-tocopherol treatment at MBDC on the biofilm destruction of treated groups compared to the NC is also shown in Fig. 3, whereby the SEM images of treated groups show a reduction in bacterial cell number (Table 2) and less compact aggregation of bacterial cells on the polystyrene surfaces.

**Table 2.** Effect of α-tocopherol on the number of bacterial cells on SEM images of *S. aureus* ATCC 6538P, *Ps. aeruginosa* ATCC 27853, and *Pr. mirabilis* ATCC 12453.

Bacteria	Bacterial cell number (log cells cm^−2^)	
	NC	Treatment
*Staphylococcus aureus* ATCC 6538P	9.65 ± 0.01^a^	9.59 ± 0.00^bA^
*Pseudomonas aeruginosa* ATCC 27 853	10.15 ± 0.00^a^	9.35 ± 0.01^bB^
*Proteus mirabilis* ATCC 12 453	9.56 ± 0.00^a^	9.44 ± 0.00^bC^

All experiments were carried out in duplicates, and the number of bacterial cells was expressed as mean log cells cm^−2^ ± standard deviation. Different lowercase letters indicate significant differences in bacterial cell number within the same bacterial strain at *P* < .05 as analysed by paired *t*-test. Different uppercase letters indicate significant differences in bacterial cell number between bacterial strains at *P* < .05, as analysed by two-way ANOVA.

## Discussion

The MIC results are consistent with previous studies (Andrade et al. 2014, Tintino et al. 2016), where the MIC values for *S. aureus* and *Ps. aeruginosa* were more than 1 mg ml^−1^ of α-tocopherol. α-Tocopherol did not exhibit antimicrobial activity from 0.01 to 2 mg ml^−1^ against *S. aureus* ATCC 6538P, *Pr. mirabilis* ATCC 12453, and *Ps . aeruginosa* ATCC 27853. To the authors’ best knowledge, the antimicrobial effect of α-tocopherol on *P. mirabilis* is not reported. Chloramphenicol was used as a PC in this broth microdilution assay. The MIC value of chloramphenicol was similar to those reported in the literature, indicating the validity of the tests (Andrews 2001, Yong et al. 2017).

α-Tocopherol was tested for its biofilm destruction activity against *S. aureus* ATCC 6538P*, Pr. mirabilis* ATCC 12453, and *Ps. aeruginosa* ATCC 27853 (Fig. 1). The concentration of α-tocopherol tested ranges from 2 to 0.01 mg ml^−1^. α-Tocopherol was able to disrupt the biofilms formed by *S. aureus* ATCC 6538P and *Pr. mirabilis* ATCC 12453 at all the concentrations of α-tocopherol tested, whereas it only disrupted *Ps. aeruginosa* ATCC 27853 biofilm at 2 mg ml^−1^ (Fig. 1). To the authors’ best knowledge, this is the first study to report on the biofilm destruction activity of α-tocopherol on *Ps. aeruginosa* ATCC 27853 and *Pr. mirabilis* ATCC 12453, which are Gram-negative bacteria.

The result obtained for *S. aureus* ATCC 6538P is in accordance with another study where α-tocopherol was reported to destroy the biofilm of *S. aureus* ATCC 6538P at 0.01 mg ml^−1^ (Mawang et al. 2017). Nonetheless, there is no significant difference in the percentage of biofilm destruction between 2 and 0.01 mg ml^−1^ α-tocopherol for *S. aureus* ATCC 6538P and *Pr. mirabilis* ATCC 12453.

*Ps. aeruginosa* biofilm is mainly composed of extracellular DNA (eDNA), with the other three exopolysaccharides (EPS), which are alginate, Psl, and Pel (Ciofu and Tolker-Nielsen 2019). The synergistic effect between the three EPS affects the biofilm architecture and formation. Pel, eDNA, and live bacterial cells establish a connected meshwork that shows increased cell-to-cell interaction. This adds up to the increased compactness of the biofilm of *Ps. aeruginosa* when compared to the biofilms of *S. aureus* and *Pr. mirabilis* (Ghafoor et al. 2011, Ciofu and Tolker-Nielsen 2019). This may explain the lower percentage of biofilm destruction of α-tocopherol towards *Ps. aeruginosa* biofilm compared to the other two bacteria tested. Nevertheless, further studies are needed to substantiate this.

α-Tocopherol was assessed for its effect on cell viability of *S. aureus* ATCC 6538P, *Pr. mirabilis* ATCC 12453, and *Ps. aeruginosa* ATCC 27853 (Fig. 2). Resazurin assay was used to determine the viability of cells within the biofilm after treatment with α-tocopherol. The non-fluorescent, blue resazurin can be reduced to fluorescent pink resorufin by cells that are metabolically active. The fluorescent intensity of resazurin assay is proportional to the number of active cells that are present (Welch et al. 2012). This enables the study of the effect of α-tocopherol on the survival of cells within the biofilm matrix, which is not reflected in the crystal violet assay.

It was observed that α-tocopherol at various concentrations tested did not show a significant reduction in the viability of *S. aureus* ATCC 6538P, *Pr. mirabilis* ATCC 12453, and *Ps. aeruginosa* ATCC 27853 (Fig. 2). This signifies that α-tocopherol does not have a killing effect on the bacteria. Instead, it disrupts the biofilm matrix to achieve the biofilm destruction effect. Without any bacteriostatic and bactericidal effect by α-tocopherol, there will be weaker selective pressure towards the bacterial cells. Therefore, this will be advantageous for α-tocopherol as an anti-biofilm agent because the bacteria are less likely to develop resistance against it (Mawang et al. 2017).

The SEM images in Fig. 3 show a reduction in bacterial cell number of *S. aureus* ATCC 6538P, *Pr. mirabilis* ATCC 12453, and *Ps. aeruginosa* ATCC 27853 (Table 2), and less compacted aggregates of cells on the polystyrene surfaces after α-tocopherol treatment when compared with the NC. These observations support the biofilm destruction activity of the crystal violet assay (Fig. 1).

Chemical cleaners such as hydrogen peroxide can have a negative impact on the users and the environment. Natural anti-biofilm compounds could be used with chemicals to reduce these adverse effects (Yong et al. 2019b). The reduction of bacterial cell number (Table 2) using α-tocopherol in mature biofilm is lower when compared to treatment with betacyanin, a natural biofilm-inhibiting agent. Betacyanin possesses biofilm inhibition activity against *S. aureus* and *Ps. aeruginosa*. It was effective in reducing 1.1–1.2 log orders of *Ps. aeruginosa* and *S. aureus* (Yong et al. 2021). The bacterial cells in a premature biofilm are more metabolically active than the dormant cells in a mature biofilm, making them more susceptible to antibiotic treatment (Shahidi and de Camargo 2016, Ghosh et al. 2020). This may be the same for biofilm inhibiting agents, as they can interfere with biofilms that are not fully developed through multiple mechanisms, such as inhibition of bacteria adhesion to a surface by interfering with the quorum-sensing system that takes part in biofilm formation, whereas biofilm destruction agents mainly rely on the disassembly of mature biofilms by interfering with EPS matrix (Shahidi and de Camargo 2016). The effect of α-tocopherol on the biofilms of the three bacteria may be due to the variation in the EPS components of these bacteria but this requires further investigation.

The α-tocopherol was shown to be able to disrupt the biofilms of *S. aureus, Pr. mirabilis*, and *Ps. aeruginosa*. As of now, there are limited studies that investigate the mechanism of the biofilm-disrupting of α-tocopherol. One commonly known mechanism in relation to biofilm disruption is the solubilization of the matrix components of biofilms, such as polysaccharides (Mawang et al. 2017, Yong et al. 2019b). As the biofilms of *S. aureus, Pr. mirabilis*, and *Ps. aeruginosa* contain polysaccharides, this could be the potential mechanism of biofilm disruption by α-tocopherol. However, further study is required to investigate the mechanism of biofilm destruction.

## Conclusions

α-Tocopherol was effective in disrupting biofilms formed by *S. aureus* ATCC 6538P (46%) and *Pr. mirabilis* ATCC 12453 (42%) at 0.01 mg ml^−1^, as well as *Ps. aeruginosa* ATCC 27853 (28%) at 2 mg ml^−1^. Further study of the dose-dependence of the biofilm destruction capability of α-tocopherol towards *S. aureus* ATCC 6538P and *Pr. mirabilis* ATCC 12453 should be conducted using lower α-tocopherol concentrations. As *Ps. aeruginosa* ATCC 27853 biofilms were only reduced at the highest concentration of 2 mg ml^−1^ α-tocopherol used, further studies should use even higher concentrations to establish the dose–response curve. As this study investigated 24 h biofilm formation, this could lead to premature biofilm conditions. Further hours of biofilm formation, such as 48 and 72 h, could be investigated in the future to provide a more comprehensive understanding of the effect of α-tocopherol on mature biofilms. As the effect of α-tocopherol on biofilms from reference strains investigated in this study is promising, clinical isolates should be investigated as they produce different biofilms. Besides, as biofilm-associated infections usually involve polymicrobial biofilms, polymicrobial biofilms can be investigated. Resazurin assay revealed that α-tocopherol does not affect the viability of cells within biofilms. Hence, it was deduced that α-tocopherol destroys biofilm by affecting the biofilm matrix only, thus degrading the structural integrity of the biofilms.

Images of scanning electron microscopy show that α-tocopherol significantly reduced bacterial cell number as well as compacted cell aggregates of *S. aureus* ATCC 6538P, *Ps. aeruginosa* ATCC 27853, and *Pr. mirabilis* ATCC 12453 on polystyrene surface. Materials for making catheters will be good choices of surfaces to investigate in the future. It is also important to investigate the mechanism for biofilm destruction of these bacteria by α-tocopherol. α-Tocopherol can potentially be a natural biofilm destruction agent against *S. aureus* ATCC 6538P, *Ps. aeruginosa* ATCC 27853, and *Pr. mirabilis* ATCC 12453.
